# Heat tolerance and acclimation capacity in subterranean arthropods living under common and stable thermal conditions

**DOI:** 10.1002/ece3.5782

**Published:** 2019-12-04

**Authors:** Susana Pallarés, Raquel Colado, Toni Pérez‐Fernández, Thomas Wesener, Ignacio Ribera, David Sánchez‐Fernández

**Affiliations:** ^1^ Marine Biology and Ecology Research Centre School of Biological and Marine Sciences University of Plymouth Plymouth UK; ^2^ Instituto de Ciencias Ambientales Universidad de Castilla‐La Mancha Toledo Spain; ^3^ Departamento de Ecología e Hidrología Universidad de Murcia Murcia Spain; ^4^ Grupo de Espeleología de Villacarrillo Jaén Spain; ^5^ Zoological Research Museum Alexander Koenig Bonn Germany; ^6^ Institut de Biologia Evolutiva (CSIC‐UPF) Barcelona Spain

**Keywords:** climate change, physiological plasticity, subterranean biology, troglobiont, upper lethal temperature

## Abstract

Cave‐dwelling ectotherms, which have evolved for millions of years under stable thermal conditions, could be expected to have adjusted their physiological limits to the narrow range of temperatures they experience and to be highly vulnerable to global warming. However, most of the few existing studies on thermal tolerance in subterranean invertebrates highlight that despite the fact that they show lower heat tolerance than most surface‐dwelling species, their upper thermal limits are generally not adjusted to ambient temperature. The question remains to what extent this pattern is common across subterranean invertebrates. We studied basal heat tolerance and its plasticity in four species of distant arthropod groups (Coleoptera, Diplopoda, and Collembola) with different evolutionary histories but under similar selection pressures, as they have been exposed to the same constant environmental conditions for a long time. Adults were exposed at different temperatures for 1 week to determine upper lethal temperatures. Then, individuals from previous sublethal treatments were transferred to a higher temperature to determine acclimation capacity. Upper lethal temperatures of three of the studied species were similar to those reported for other subterranean species (between 20 and 25°C) and widely exceeded the cave temperature (13–14°C). The diplopod species showed the highest long‐term heat tolerance detected so far for a troglobiont (i.e., obligate subterranean) species (median lethal temperature after 7 days exposure: 28°C) and a positive acclimation response. Our results agree with previous studies showing that heat tolerance in subterranean species is not determined by environmental conditions. Thus, subterranean species, even those living under similar climatic conditions, might be differently affected by global warming.

## Graphical abstract

Heat tolerance and acclimation capacity were studied in four subterranean species of distant arthropod groups but living under similar selection pressures (exposed to the same constant environmental conditions for a long time). Upper thermal limits widely exceeded ambient temperature and differed among the species, as well as acclimation responses. Our results show that heat tolerance in subterranean species is not determined by environmental conditions. Cave species, even those which live under similar climatic conditions, might be differently affected by global warming.

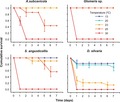

## 1 INTRODUCTION

According to Janzen's mountain passes hypothesis (Janzen, 1967) and the climatic variability hypothesis (Stevens, 1989), a positive relationship exists between the thermal tolerance breadth and the level of climatic variability experienced by taxa, which has been demonstrated at different taxonomic resolutions (e.g., Addo‐Bediako, Chown, & Gaston, 2000; Calosi, Bilton, & Spicer, 2007; Cruz, Fitzgerald, Espinoza, & Schulte, 2005; Kellermann, Heerwaarden, Sgrò, & Hoffmann, 2009; Sunday, Bates, & Dulvy, 2011). Similarly, species from stable environments are predicted to have a lower thermal acclimation capacity than those from more variable habitats (e.g., Feder, 1978; Markle & Kozak, 2018; Shah, Funk, & Ghalambor, 2017; Tomanek, 2010 and references herein). As an extreme example, some Antarctic cold‐stenothermal species (e.g., Clark, Fraser, Burns, & Peck, 2008; Clark Fraser & Peck, 2008; La Terza, Papa, Miceli, & Luporini, 2001; Rinehart et al., 2006; Somero, 2005) as well as warm stenothermal coral reef fishes (Kassahn et al., 2007; Nilsson, Östlund‐Nilsson, & Munday, 2010) have lost the ability to activate a heat shock response via the expression of heat shock proteins, which makes them especially vulnerable to global change (Kellermann & van Heerwaarden, 2019; Patarnello, Verde, Prisco, Bargelloni, & Zane, 2011; Somero, 2005). These questions remain poorly explored in other extremely thermally stable habitats, such as subterranean environments, although some recent studies have found support for the climatic variability hypotheses for cave springtails (Raschmanová, Šustr, Kováč, Parimuchová, & Devetter, 2018) and spiders (Mammola, Piano, Malard, Vernon, & Isaia, 2019). However, the generality of such patterns remains controversial (see Gunderson & Stillman, 2015; Rohr et al., 2018), as other studies have found weak support for a correlation between some thermal tolerance traits and the magnitude or predictability of thermal variability (e.g., Kellermann et al., 2012; Overgaard, Kearney, & Hoffmann, 2014; Seebacher, White, & Franklin, 2015).

Subterranean species are generally assumed to be stenothermal (i.e., to have thermal tolerance ranges adjusted to the narrow range of temperatures they experience in their habitat), since thermal stability in these climatically buffered environments could impose a strong selective pressure on thermal tolerance traits (Angilletta, 2009; Culver & Pipan, 2009; Humphreys, 2018). In addition, specialist species may lack genetic variation in key traits, limiting their ability to adapt to conditions beyond their current range (Kellermann et al., 2009). The mechanisms to cope with heat stress may also entail high metabolic costs, difficult to assume in resource‐limited habitats such as the subterranean environment (Angilletta, 2009; Huey & Slatkin, 1976). The heat shock response, for example, is energetically costly, and it has been shown to be maladaptive when conditions are not stressful (Krebs & Loeschcke, 1994; Tomanek, 2010), but such costs have not been explored in cave‐dwelling organisms.

However, several decades ago, Vandel (1965) questioned the strict stenothermal character of cave fauna, based on the little experimental data available at that time for some cave‐adapted invertebrates. Such data showed that they generally survive relatively long‐term exposures at 20°C (much higher than the usual temperatures of their habitats) and can resist up to 25–29°C for short exposures (e.g., Beauchamp, 1932; Ginet, 1960; Glaçon, 1953; Szymczkowsky, 1953). Mitchell (1971) also reported that the carabid *Rhadine subterranea* (Van Dyke, 1919) shows a seasonal shift in its temperature preference, showing some degree of thermal plasticity. Bull and Mitchell (1972) demonstrated that the cave millipedes *Cambala speobia* (Chamberlin, 1952) and *Speodesmus bicornourus* Causey, 1959 could survive 30°C (although only for some hours), despite living at constant temperatures close to 20°C. Experimental data on thermal tolerance in subterranean ectotherms are still very scarce compared with those on surface‐dwelling (i.e., epigean) species, which in part could be due to the difficulties in collecting large number of specimens required for experiments or rearing them in the laboratory (Mammola, Cardoso, et al., 2019; Raschmanová et al., 2018). The relatively scarce, more recent studies that have specifically measured thermal breadth and plasticity in subterranean species show contrasting results for different taxa. Some species present the typical characteristics of stenothermal organisms, that is, low physiological plasticity (e.g., Di Lorenzo & Galassi, 2017) and narrow thermal breadths for survival (e.g., some deep subterranean spiders – Mammola, Piano, Malard, et al., 2019), locomotion or respiration (e.g., Issartel, Hervant, Voituron, Renault, & Vernon, 2005; Mermillod‐Blondin et al., 2013), being sensitive to changes of only a few grades below or above their habitat temperature. In contrast, other subterranean species have shown much wider thermal ranges for performance and survival (Issartel et al., 2005; Mammola, Piano, Malard, et al., 2019; Mermillod‐Blondin et al., 2013). Studies on subterranean leiodid beetles have reported similar thermal tolerance ranges (between 0 and 20–23°C) among different species irrespective of the climatic conditions of the caves where they live (Glaçon, 1953; Juberthie et al., 1984; Rizzo, Sánchez‐Fernández, Fresneda, Cieslak, & Ribera, 2015), suggesting a lack of adjustment of thermal limits to environmental conditions in this group. What is common from these studies is that subterranean ectotherms have lower heat tolerance limits than most epigean species (not higher than 25–26°C in the long‐term), as expected considering habitat thermal variability. However, a notable number of subterranean species can tolerate temperatures much higher than their current habitat conditions or even those experienced through their evolutionary history (e.g., Rizzo et al., 2015). This suggests that heat tolerance could be to some degree conserved across subterranean ectotherm taxa, in line with the general trend in surface‐dwelling ones, for which plastic and evolutionary changes in upper thermal limits seem to be highly constrained (Araújo et al., 2013; Hoffmann, Chown, & Clusella‐Trullas, 2013). However, most data on thermal tolerance for cave‐dwelling organisms come from unrelated, disparate species from different geographical areas (and therefore living under different climatic conditions), which makes it difficult to determine to what extent interspecific variation in thermal tolerance is phylogenetically constrained or environmentally‐driven.

To explore heat tolerance and acclimation capacity in subterranean ectotherms by controlling for the potential influence of different thermal history, we focused on different unrelated taxa that have been exposed to the same environmental conditions for a long time. We studied heat tolerance, accounting for both basal (upper lethal temperature—ULT) and induced tolerance (plasticity of ULTs via acclimation), through controlled laboratory experiments, in four distantly related arthropod species with different evolutionary origins that inhabit the same cave.

## 2 MATERIALS AND METHODS

### 2.1 Target species, sampling, and holding conditions

The four studied species coexist in “Murcielaguina de Hornos,” a cave with two entrances of 5,135‐m extent and 80‐m depth located in Jaén, Spain (latitude 38.2158200996, longitude −2.7111768723, and altitude 1,081 m.a.s.l.) (Pérez‐Fernandez, Pérez‐Ruiz, & Pérez‐Fernandez, 2016). The target species belong to three different orders of arthropods: (a) *Glomeris* sp. (Diplopoda: Glomerida: Glomeridae) is an undescribed species of a mega‐diverse genus within the order Glomerida (Golovatch, Mauriès, Akkari, Stoev, & Geoffroy, 2009) confined to some caves in South Spain (unpublished observations), which shows well‐developed troglomorphic traits (depigmentation, reduced thickness of tergites, reduced ocelli, and elongation of body and appendages, see Liu, Golovatch, Wesener, & Tian, 2017); (b) *Deuteraphorura silvaria* (Gisin, 1952) (Entognatha: Collembola: Onychiuridae) is distributed in Central and South Europe (Arbea, 2003, 2013); (c) *Speonemadus angusticollis* (Kraatz, 1870) (Coleoptera: Leiodidae) is an Iberian endemic species, and (d) *Atheta subcavicola* (Brisout de Barneville, 1863) (Coleoptera: Staphylinidae) is one of the few species of the genus *Atheta* associated with caves (Assing & Vogel, 2003), distributed in the Iberian Peninsula and France (Balazuc, Miré, Sigwalt, & Théodoridès, 1951; Barranco et al., 2004; Reboleira, Gonçalves, & Oromí, 2011). The four species are usually found close to guano deposits in the study cave, but show different degrees of subterranean specialization. *Glomeris* sp. is the only troglobiont species among the studied ones (i.e., strictly subterranean sensu Sket, 2008), as it has been never found outside caves and it is always found in the deeper parts (>50 m from the entrance) of the Murcielaguina cave. *Speonemadus angusticollis* as a species cannot be considered as a true troglobiont because its populations in the central part of the Iberian Peninsula are endogean, that is, live in the soil or plant litter (Fresneda, 2013). In fact, although it has elongated body and appendages, this species does not show other typical morphological traits of cave‐adapted species such as a lack or reduction of eyes and wings or depigmentation (Fresneda, Grebennikov, & Ribera, 2011; Perreau, 2009; Reboleira, Fresneda, & Salgado, 2017). However, in its southern distribution (Andalusia region, including the present study site), *S. angusticollis* has been only found in caves, generally closer to the entrances. The other two species studied here are cave‐facultatives (i.e., nonobligate subterranean species), as they can be also found in the soil or plant litter outside the cave.

Adults of the four species were collected by hand on June 2018 inside the cave at approx. 50 m (the Collembola *D. silvaria*) or 60 m from the entrance (the rest of the species). Temperature at sampling was 13°C and humidity 93% RH in both sites (measured with a digital thermohygrometer, OM‐EL‐USB‐2‐LCD; Omega Engineering, Seville, Spain). Specimens were transported to the laboratory in the “Instituto de Ciencias Ambientales (ICAM)” of the University of Castilla‐La Mancha (Toledo, Spain) under controlled, cold, and humid conditions to minimize stress. Substrate from the cave and moss was used to maintain a high humidity (>90% RH) during transport. In the laboratory, the specimens were acclimatized for 2 days before the experiments in plastic containers (10 × 15 cm) with a white plaster layer (approx. 1cm), cave substrate and wetted tissue paper, at the same temperature than the cave (13°C) in an incubator (Radiber ERF‐360). The containers were closed with plastic film with small holes for aeration. To avoid any potential stress caused by starvation or desiccation, food was provided ad libitum with freshly frozen *Drosophila melanogaster* throughout the entire duration of the experiments and humidity was always maintained at levels close to saturation by wetting the containers and tissue paper daily and placing trays with distilled water inside the incubators.

For the experiments, specimens of *Glomeris* sp., *A. subcavicola*, and *S. angusticollis* were placed in the plastic containers as previously described. As *D. silvaria* specimens were not easily visible in such containers given their small size, they were placed in Petri dishes also with a plaster substratum and a tissue paper saturated in water. Temperature and relative humidity inside the plastic containers were recorded at 5‐min intervals with HOBO MX2301 dataloggers (Onset Computer Corporation).

### 2.2 Basal heat tolerance: upper lethal temperatures

**Figure 1:**
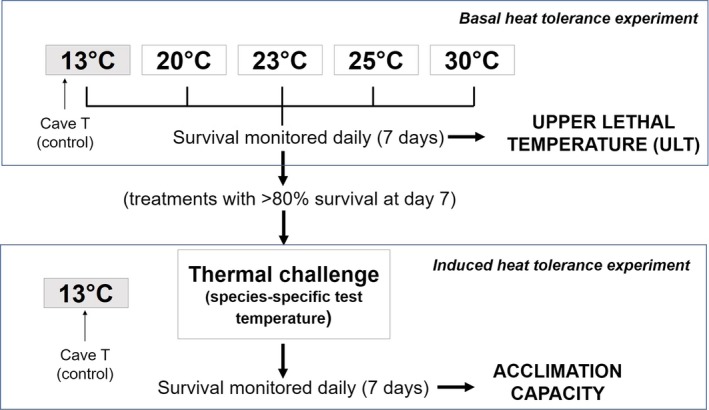
Schematic representation of the experimental design

To estimate species' heat tolerance, we assessed survival at different temperatures during long‐term exposure (7 days) (Figure 1). The experimental containers with the specimens (*N* = 7–16 individuals per species and treatment) were placed in Radiber incubators at 13 (control temperature equal to that of the cave), 20, 23, 25 or 30°C and >90% RH. Survival was checked every 24 hr and specimens were recorded as alive if they were capable of some movement after a slight touch with a brush.

### 2.3 Induced heat tolerance: acclimation capacity

Acclimation capacity was assessed using specimens (*N* = 8–16 individuals per species and treatment) from the heat tolerance experiment (i.e., previously exposed at the different temperatures or pretreatments hereafter), which were subsequently exposed to a thermal challenge at a high temperature (test temperature hereafter). Only those pretreatments in which all or most of the individuals had survived during the entire duration of the basal heat tolerance experiment (>80% survival after 7 days) were used to determine acclimation capacity (Figure 1). The test temperature was set up considering the specific survival response in the previous basal heat tolerance experiment. Those species which showed a higher basal heat tolerance, that is, >50% survival after 7 days in the 25°C pretreatment (*Glomeris* sp., *A. subcavicola*, and *S. angusticollis*) were transferred to 30°C, while those with lower survival rates (*D. silvaria*) were transferred to 23°C. Once transferred from the pretreatments to the test temperature, survival was checked every 24 hr for another 7 days. During such time, groups of five individuals of each species were kept at the cave temperature (13°C) to control for potential mortality associated with uncontrolled factors other than thermal stress.

### 2.4 Data analyses

All the analyses were performed in R v.3.3.3. To explore species' basal heat tolerance, survival at the different temperatures (basal heat tolerance experiment) was compared using Kaplan–Meier survivorship curves (Altman, 1992). Right censored data were specified for those individuals that were alive at the end of this experiment, that is, after 7 days (see Therneau, 2015). Survival data from each species at day seven were fitted to a logistic regression model from which LT_50_ (median lethal temperature, that is, the temperature at which 50% of the exposed individuals have died) were estimated using the *dose.p* function.

To determine acclimation capacity, we tested for the effect of acclimation temperature (i.e., pretreatments from the basal heat tolerance experiment) on survival time during the subsequent thermal challenge. For this, we fitted GLMs assuming a Poisson distribution, separately for each species. When a significant effect of acclimation temperature (pretreatment) was found, post hoc tests with Bonferroni correction were used to compare pairs of pretreatments and specifically to test for positive acclimation responses (i.e., significantly higher survival in individuals from the different heat pretreatments than those from the control pretreatment – 13°C) or detrimental acclimation effects (i.e., significantly lower survival in individuals from heat treatments than those of the control pretreatment). Additionally, we employed analogous nonparametric tests (Kruskal–Wallis) and *kruskalmc* posthoc test (Giraudoux, 2016; Siegel & Castellan, 1988) for comparison with the previous, more conservative approach, and to test for the robustness of the results.

## 3 RESULTS

### 3.1 Basal heat tolerance

**Figure 2:**
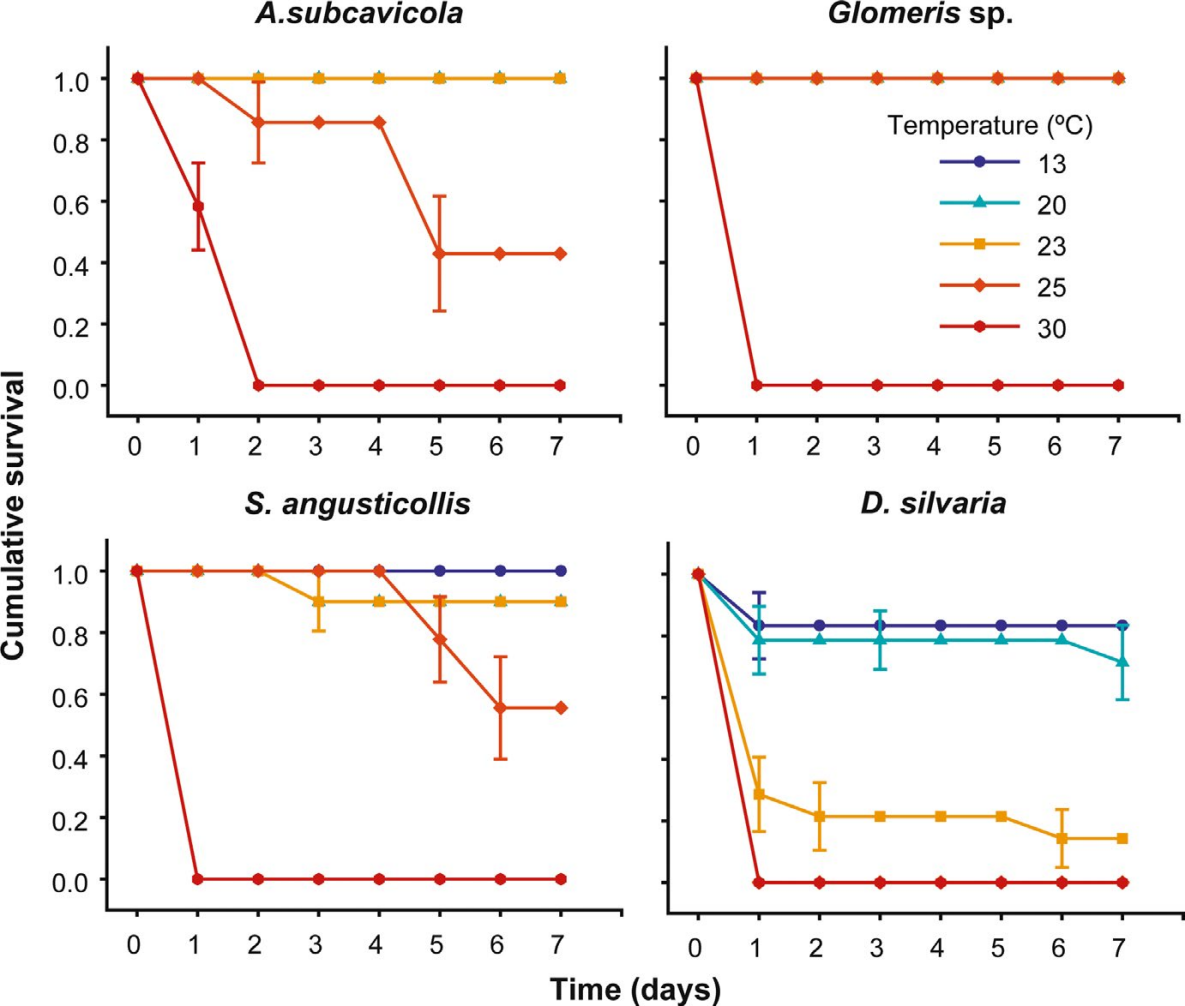
Kaplan–Meir survivorship curves for each temperature treatment used to measure upper lethal limits. Each data point represents survival probability (mean ± *SEM*)

All the species showed ca. 100% survival at 13°C (cave temperature) and 20°C during the seven exposure days and a rapid mortality (between 24 and 48 hr) at 30°C. Therefore, survival at 23 and 25°C marked the differences in basal heat tolerance among the species: *Glomeris* sp. showed the highest survival at these temperatures (100% survival for 7 days) and *D. silvaria* the lowest (i.e., rapid and high mortality in both treatments) (Figure 2). Mean LT_50_ values (7 days) ranged from 19.64 ± 1.03 (*D. silvaria*), 24.98 ± 0.52 (*A. subcavicola*), 25.18 ± 0.75 (*S. angusticollis*) to 27.59 ± 0.76°C (*Glomeris* sp.).

### 3.2 Acclimation capacity

**Figure 3:**
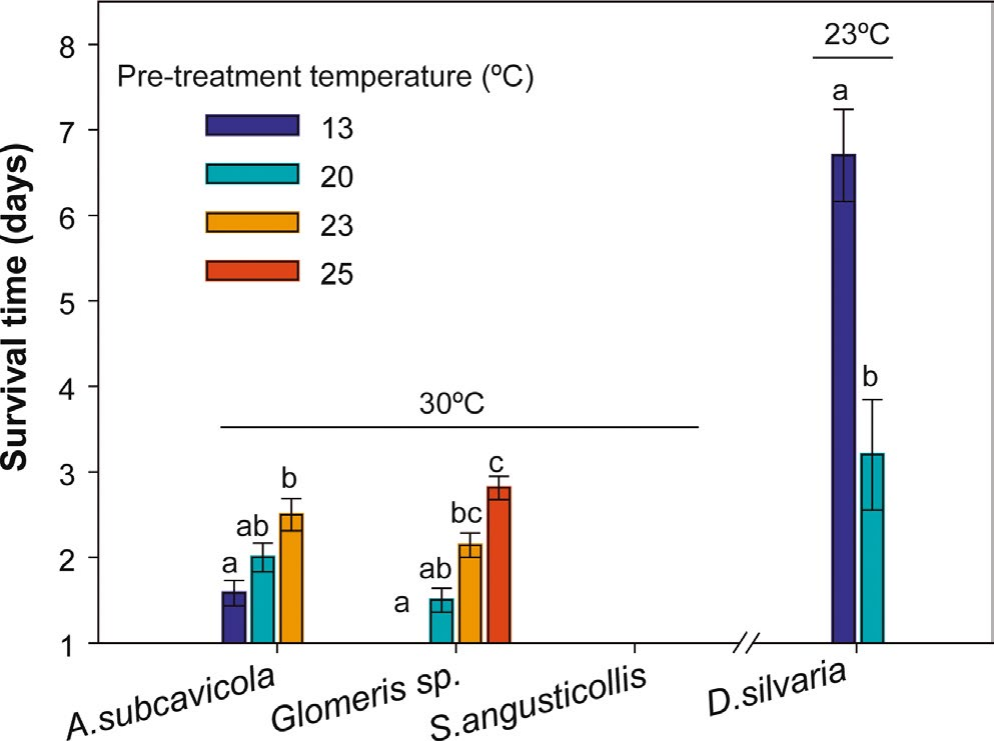
Survival time at a fixed temperature (indicated above each species) after previous acclimation at different temperatures (represented by different colors in the legend). Letters above the bars indicate significant differences among acclimation treatments within species according to post hoc tests (*p* < .05). Survival time for *Speonemadus angusticollis* is not shown because it was lower than 1 day in all treatments

Each species showed a different response after acclimation at the different temperatures and the subsequent thermal challenge. *Glomeris* sp. and *A. subcavicola* showed a positive acclimation response, although in the latter only nonparametric tests detected significant differences among acclimation treatments (Table 1). In *Glomeris* sp., survival time at 30°C was significantly higher in individuals previously acclimated at 23 and 25°C than those acclimated at the cave temperature (control, 13°C), with a maximum difference in mean survival time of 1.8 days between 25°C and 13°C. In *A. subcavicola,* only those individuals acclimated at 23°C showed a significantly higher survival time (1 day) than those from the 13°C pretreatment (*p* < .05 in *kruskalmc* post hoc test) (Figure 3). *Speonemadus angusticollis* had no acclimation capacity: no specimens of any of the acclimation treatments survived more than 1 day of exposure at 30°C. In *D. silvaria,* previous acclimation at 20°C significantly reduced subsequent tolerance to 23°C (Figure 3).

**Table 1:** Results of GLM and Kruskal–Wallis tests to determine the effect of acclimation temperature on the subsequent survival at a fixed temperature

Species	GLM (Poisson)			Kruskal–Wallis test		
	*X* ^2^ value	*df*	*p* value	*X* ^2^ value	*df*	*p* value
*Atheta subcavicola*	3.798	2	.362	10.279	2	.006
*Glomeris* sp.	14.496	3	.002	40.137	3	<.001
*Speonemadus angusticollis*	–	–	–	–	–	–
*Deuteraphorura silvaria*	12.645	1	<.001	9.405	1	.002

No mortality was recorded in the control groups (i.e., individuals maintained at 13°C for the entire duration of both experiments).

## 4 DISCUSSION

As expected, upper thermal limits of the studied species were lower than those of most surface‐dwelling organisms (Bennett et al., 2018). However, the four species studied here can survive, at least for 1 week, at temperatures much higher than the narrow range currently experienced in their habitat (ca. 13‐14°C). These results are in agreement with previous work showing that many subterranean species can withstand temperatures up to 20°C with no apparent signs of stress (e.g., Issartel et al., 2005; Mermillod‐Blondin et al., 2013; Rizzo et al., 2015). Although narrower thermal limits have been reported for some cave terrestrial invertebrates (e.g., Mammola, Piano, Malard, et al., 2019), it seems that such limits are generally not adjusted to ambient temperature in subterranean ectotherms, in contrast with taxa from other thermally stable (but also much colder) environments such as polar waters (Peck, Webb, & Bailey, 2004; Somero & DeVries, 1967). Such higher thermal sensitivity of polar species might be related to the different mechanisms for thermoregulation in aquatic species or the role of oxygen in setting thermal tolerance, which is particularly relevant for aquatic organisms (Verberk et al., 2016).

Surprisingly, the diplopod *Glomeris* sp., the only true troglobiont (i.e., obligate subterranean) species in our study, showed an extraordinarily high heat tolerance (mean 7 days‐LT_50_ of 27.6°C) and a capacity to survive for more than 24 hr at 30°C. Although experimental data on thermal physiology are still scarce for subterranean ectotherms—and particularly for cave diplopods (but see Bull & Mitchell, 1972), to our knowledge, no known troglobiont species studied so far have shown such a tolerance to high temperature for long‐term exposures. Nevertheless, thermal breadths for critical biological functions important to long‐term survival or reproduction (e.g., feeding, locomotion, fecundity) may be more constrained than survival ranges in subterranean species (e.g., Mermillod‐Blondin et al., 2013). Similarly, exposure to nonlethal temperatures may have “hidden costs” through sublethal effects (e.g., oxidative stress) which could affect fitness in the longer term (Monaghan, Metcalfe, & Torres, 2009). It should be also noted that earlier stages could display different thermal sensitivity than adults. For example, data on the development of some strictly subterranean leiodid species showed high egg mortality between 15 and 21°C (Delay, 1978; Juberthie et al., 1984).

Other than absolute lethal limits, we also found differences in the degree of plasticity among the studied species. In comparative terms, thermal plasticity ranged from high in *Glomeris* sp. to moderate in *A. subcavicola* and absent in *S. angusticollis* and *D. silvaria*. Taken together, these results suggest that maintaining heat tolerance beyond the range of temperatures experienced, as well as some degree of thermal plasticity, could be less costly than a priori expected in a constant, stable, and resource‐limiting environment such as caves. Caves are environments with extreme selection pressures and adapted organisms that are similar in appearance, physiology, and behavior all over the world, despite their different origins (Howarth & Moldovan, 2018; Liu et al., 2017; Trontelj, Blejec, & Fišer, 2012). However, our results show that upper thermal limits and acclimation capacities are not so homogeneous across subterranean invertebrates cohabiting the same cave, suggesting a weak role of thermal stability as a selective pressure on heat tolerance traits. Instead, heat tolerance seems to be linked to particular aspects of the ecology, physiology, or evolutionary history of the different lineages.

The degree of specialization to the subterranean environment, as well as habitat selection within the cave (both aspects related to the thermal variability to which organisms are exposed) have been associated with differences in the thermal tolerance among subterranean species. Some troglobionts have been shown to be less thermotolerant than facultative cave‐inhabitants (e.g., Raschmanová et al., 2018). Such relationship was also consistent across a set of 37 phylogenetically distant invertebrate species (Novak et al., 2014). Within troglobionts, species confined to the internal parts of the caves show also higher thermal sensitivity than closely related ones found closer to cave entrances, where thermal conditions are more fluctuating (Latella, Bernabò, & Lencioni, 2008; Mammola, Piano, Malard, et al., 2019). Our small set of unrelated species and the lack of phylogenetic information of the studied lineages prevent us to test this relationship between the degree of subterranean specialization and thermal tolerance within an appropriate phylogenetic context. However, it is remarkable that, in contrast with these previous studies, the highest upper thermal limits and acclimation capacity in our study were displayed by a troglobiont species which is only found in the most internal part of the cave Murcielaguina de Hornos (*Glomeris* sp.). In contrast, the other species, which can be found closer to the cave entrance (*S. angusticollis*) or are not obligate subterranean species (*A. subcavicola* and *D. silvaria*), were more heat susceptible. The habitat preferences of these species might change across their distribution range, as in *S. angusticollis* (Fresneda, 2013); therefore, it would be also interesting to explore intraspecific differences on thermal tolerance related with the degree of specialization to the subterranean environment.

The differences in thermal physiological limits observed here among the studied species seem to reflect the specific evolutionary history of each lineage rather than distinct environmental preferences related to the current habitat. If thermal tolerance breadths have been progressively reduced in the process of specialization to underground environments, one might expect that lineages which have been isolated in caves for a longer time would show the most modified thermal tolerance ranges (i.e., most reduced with respect to that of the closest surface‐dwelling ancestor), assuming a paradigm of evolution of thermal physiology traits similar to that of time‐correlated troglomorphies (Culver, Holsinger, Christman, & Pipan, 2010; Ribera, Cieslak, Faille, & Fresneda, 2018). Unfortunately, we still lack both molecular and physiological data needed to track the evolutionary reduction in thermal tolerance in the process of colonization of subterranean habitats. However, our results with *Glomeris* sp. suggest that this species may still retain some heat tolerance from a relatively recent epigean ancestor. Unpublished barcoding data suggest a close relationship of the cave species to *Glomeris maerens* Attems, 1927 (ca. 10% uncorrected p‐distance), an ecologically flexible species often found in semi‐open habitat such as Mediterranean shrub and evergreen forests, at sea levels up to 1,700‐m elevation (Kime & Enghoff, 2011), but additional molecular data are needed to support such hypothetically close phylogenetic relatedness. Despite showing other well‐developed troglomorphic traits (depigmentation, reduced ocelli, elongation of body and appendages, see Liu et al., 2017), this species might belong to a lineage which colonized caves more recently than the other, less troglomorphic species studied here. This also raises another interesting question for future research, as the asynchronous evolution of morphological and physiological traits in subterranean fauna.

Accurate predictions of species responses to climate change are mandatory if we aim to develop effective management strategies to face this problem. In summer, maximum surface temperatures in the study site exceed or are very close to the ULTs of the species studied here. Although warming will be buffered and delayed in subterranean systems (Domínguez‐Villar, Lojen, Krklec, Baker, & Fairchild, 2015), these will not escape from the effects of climate change (Mammola, Goodacre, & Isaia, 2017; Mammola, Piano, Cardoso, et al., 2019). We show here that subterranean species, even those living under the same climatic and stable conditions, might be differently affected by global warming. Among the studied species, the population of the collembolan *D. silvaria* in the Murcielaguina cave, at the warmest edge of its distribution range (Arbea, 2003, 2013), might be particularly threatened considering its low heat tolerance.

Our results stress the need of experimental approaches to assess the capability of species to cope with temperatures outside those they currently experience and give rise to further research questions to be explored in subterranean ecosystems, highlighting their potential as natural laboratories to study multiple eco‐evolutionary processes (Mammola, 2018; Sánchez‐Fernández et al., 2016, 2018).

## CONFLICT OF INTEREST

The authors have no competing interest to declare.

## AUTHOR CONTRIBUTIONS

S.P. and D.S.‐F. conceived the idea and designed the experiments. T.P.‐F. collected the specimens. S.P., R.C., and D.S.‐F. performed the experiments. T.P.‐F., T.W., and I.R. provided background information on species ecology and evolution. S.P. analyzed the data and led the manuscript writing. All authors discussed the results, contributed to the manuscript drafts, and gave final approval for publication.

## Data Availability

Raw data from the experiments conducted in this study are available in the publicly accessible repository FigShare (https://doi.org/10.6084/m9.figshare.9948467).
